# Effect of concurrent repair of medial meniscal posterior root tears during high tibial osteotomy for medial osteoarthritis during short-term follow-up: a systematic review and meta-analysis

**DOI:** 10.1186/s12891-021-04499-9

**Published:** 2021-07-15

**Authors:** Shin Kyun-Ho, Ryoo Hyun-Jae, Jang Ki-Mo, Han Seung-Beom

**Affiliations:** Department of Orthopedic Surgery, Anam Hospital, Korea University College of Medicine, 73 Inchon-ro, Sungbuk-gu, Seoul, 02841 South Korea

**Keywords:** Knee, Osteoarthritis, Medial meniscus, Osteotomy, Arthroscopy, Systematic review, Meta-analysis

## Abstract

**Background:**

Medial meniscal posterior root tears (MMPRTs) are frequently associated with medial compartment osteoarthritis, leading to loss of meniscal hoop tension. This study aimed to evaluate the efficacy of concurrent MMPRT repair during high tibial osteotomy (HTO) compared to HTO alone in patients with medial osteoarthritis and MMPRTs.

**Methods:**

The MEDLINE/PubMed, EMBASE, and Cochrane Library databases were searched for studies reporting on concurrent MMPRT repair during HTO. Pre- and postoperative data were pooled to investigate the treatment effects of concurrent MMPRT repair during HTO, and compare postoperative clinical, radiological, and arthroscopic outcomes including cartilage status and healing event rates according to the arthroscopic classification of MMPRT healing (complete, partial [lax or scar tissue], or failed healing) between HTO patients with and without concurrent MMPRT repair. The random-effect model was used to pool the standardized mean differences, odds ratios (ORs), 95% confidence intervals (CIs), and event rates.

**Results:**

Seven patient subgroups in six articles divided according to meniscal repair techniques were included in the final analysis. Concurrent MMPRT repair during HTO significantly improved the Lysholm score, while no intergroup differences were observed in the postoperative Lysholm and WOMAC scores, as well as radiological and arthroscopic outcomes. Those who underwent concurrent MMPRT repair showed a higher rate of complete meniscal healing (OR: 4.792, 95% CI, 1.95–11.79), with a pooled rate of complete meniscal healing of 0.327 (95% CI, 0.19–0.46).

**Conclusion:**

Concurrent MMPRT repair during HTO for medial osteoarthritis with MMPRTs has little benefits on the clinical, radiological, and arthroscopic outcomes during short-term follow-up. Further accumulation of evidence is needed for long-term effects.

## Background

Medial meniscal posterior root tears (MMPRTs) are frequently associated with medial compartment osteoarthritis (OA), as loss of meniscal hoop tension increases the contact pressure in the medial compartment [1–3]. Arthroscopic MMPRT repair has been proposed to restore meniscal hoop tension and decelerate medial tibiofemoral articular cartilage degeneration. However, the varus deformity of the lower limbs, commonly observed in medial knee OA, is an important prognostic factor in meniscal healing and long-term outcomes following MMPRT repair [4–11]. Furthermore, only MMPRT repair cannot successfully decompress one-sided medial compartment overload without correction of the varus deformities of the lower limbs.

Medial open-wedge high tibial osteotomy (HTO) is a joint preservation surgery for medial compartment OA with varus malalignment [12–15]. HTO transfers the weight-bearing line that is deviated to the medial compartment, thereby increasing the medial proximal tibial angle and reducing medial compartmental pressure. Coverage of denuded articular cartilage and prevention of OA progression can be expected after HTO [16–19]. However, the resulting fibrous cartilage quality may not be as good as that of the original hyaline cartilage [20]. Furthermore, second-look arthroscopic findings after HTO alone demonstrated that the rate of complete healing of MMRPTs was low, and most of the healed MMPRTs showed lax healing with scars according to the arthroscopic visual classification of MMPRT healing [21–23].

The long-term survival of HTO for medial OA is not guaranteed, with reported 10-year survival rates ranging from 56 to 79% [24–30]. As joint preservation surgeries aim to delay the time to total knee arthroplasty, concurrent MMPRT repair during HTO may be a logical approach to prevent OA progression by restoring medial meniscal hoop tension and the tibiofemoral contact surface.

Despite favorable outcomes following concurrent MMPRT repair during HTO, no randomized controlled studies or large-scale cohort studies have been published [31, 32]. To clarify the treatment effects using objective numerical values, a systematic review and meta-analysis of all available case series or comparative studies on concurrent MMPRT repair during HTO is required. The present study hypothesizes that concurrent MMPRT repair during HTO would improve the clinical, radiological, and arthroscopic outcomes compared to HTO alone in patients with medial OA and MMPRTs.

## Methods

This study followed the Preferred Reporting Items for Systematic reviews and Meta-Analyses (PRISMA) guidelines [33]. Patient consent and ethical approval were not required according to the study design. Two independent reviewers performed the literature search, inclusion, data extraction, and quality assessment. Disagreements were resolved with a third independent reviewer.

### Search strategy

All relevant articles were obtained from MEDLINE/PubMed, Cochrane Central Register of Controlled Trials, and EMBASE from inception to August 2020.

The following search terms, including their equivalent Medical Subject Headings (MeSH) terms, and their combinations were searched in the [Title/Abstract] field of the search engines: “knee” OR “knees” OR “tibia” OR “tibias OR “tibial” OR “tibiae” OR “knee” [MeSH term] AND “osteotomy” OR “osteotomies” OR “osteotomy” [MeSH term] AND “meniscus” OR “meniscal” OR “meniscus” [MeSH term]. No other restrictions, including language restrictions, were imposed. Relevant eligible references in the selected articles were reviewed to identify the relevant articles that were not identified during the database search.

### Eligibility criteria and study selection

Two independent reviewers screened all the titles and abstracts. Full-text screening was done on articles that showed discrepancies. Suitable studies were selected based on following inclusion criteria: a case series or comparative study reporting a clinical, radiological, or arthroscopic outcome of HTO with concurrent MMPRT repair in patients with radiograph findings of medial OA and MRI or arthroscopic findings of MMPRTs. The exclusion criteria were as follows: (1) review/technical papers and (2) inaccessible data or full text. The inter-reviewer reliability was assessed using the kappa statistic (κ). These selections were then reviewed by a third author, and discrepancies were resolved by discussion.

### Data extraction

Two authors extracted data from all selected studies. The inter-reviewer reliability was assessed using κ. Disagreements were resolved through a consensus with a third author. For comparative studies on different repair techniques, data from each technique were separated and extracted as a subgroup. The data were extracted according to the following descriptive information: (1) study characteristics, including author names, year of publication, study design, level of evidence, and journal; (2) patient demographics, such as number of cases, mean age, and sex; (3) mean follow-up period; (4) details of the surgical procedures, such as osteotomy type and MMPRT repair technique; and (5) outcomes of interest. The outcomes of interest included the following: (1) clinical and functional outcomes of knee joints, indicated by the Lysholm score [34], International Knee Documentation Committee subjective knee (IKDC) score [35], Tegner Activity Scale [35], Western Ontario and McMaster University (WOMAC) score [35], Knee Society Knee (KSKS) and Functional (KSFS) scores [36], and Hospital for Special Surgery (HSS) knee scores [37]; (2) radiological findings, including the mechanical femorotibial angle (FTA) [38], weight-bearing line ratio (WBLR) [38], width of the medial joint space (WMJS) [39], and Kellgren–Lawrence (K-L) grades [40]; (3) arthroscopic visual classification of MMPRT healing (complete, partial [lax or scar tissue], or failed healing) [21–23]; (4) amount of medial meniscal extrusion (MME) [41]; and (5) articular cartilage status assessed using the Outerbridge [42] or International Cartilage Repair Society (ICRS) grading system [43, 44]. The ICRS graded articular cartilage degeneration as follows: 1, superficial lesions, such as fissures and cracks; 2, lesions extending down to < 50% of the cartilage depth; 3, lesions extending down to > 50% but not involving the subchondral bone; and 4, lesions involving the subchondral bone. The ICRS graded articular cartilage regeneration as follows: 1, complete or nearly complete coverage of the original lesion (excellent recovery); 2, ≥ 50% coverage (good recovery); and 3, < 50% coverage (poor recovery).

Changes in outcomes were defined as postoperative-preoperative values in outcome measurements. Disagreements in the collected data were resolved through data accuracy cross-checking.

### Quality assessment

Two authors assessed the quality of all included studies. The inter-rater reliability was assessed using κ. Disagreements were resolved through discussion and consensus with a third author. The Newcastle–Ottawa assessment scale was used to assess the methodological quality of comparative studies [45, 46]. It consists of three main domains: selection, with four subdomains; comparability, with one subdomain; and outcome, with three subdomains. A study was awarded a maximum of one star for each item in the selection and outcome domains. A maximum of two stars was assigned for comparability: one for controlled age and another for controlled variables including sex, body mass index, or preoperative K-L grade [27, 47]. A total of ≥ 7 stars indicated a low-risk study, 4–6 stars indicated a moderate-risk study, and < 4 stars indicated a high-risk study.

As suggested by the Cochrane Effective Practice and Organisation of Care Group for all interrupted time-series studies, we used the seven standard criteria for methodological quality assessment as follows [48]: (1) independence, (2) pre-specification of the intervention effect, (3) effect of the intervention on data collection, (4) knowledge of allocated intervention, (5) incomplete outcome data, (6) selective outcome reporting, and (7) other risks of bias. The risk for each criterion was categorized as low, high, or unclear.

### Statistical analysis

All data from the studies were extracted using an Excel spreadsheet (Microsoft Corporation, Redmond, WA, USA). Results between the case and control groups were analyzed using R version 3.1.1 (The R Foundation for Statistical Computing). Statistical significance was set at P < 0.05. For comparative studies analyzing outcomes between various repair techniques, the data were broken down within each individual study according to each repair technique and pooled as separate subgroups in meta-analyses. The data needed to be standardized before analyses and comparison of the outcomes because of heterogeneity between the materials and methods used in the included studies. The standardized mean difference (SMD), defined as the difference in pre- and postoperative mean outcomes divided by the standard deviation of the difference in the outcome [49], was determined from both case series and comparative studies as the “best estimate” of the expected mean treatment effect of concurrent MMPRT repair during HTO. The SMD between groups was also determined for intergroup comparison of the postoperative outcomes. Meta-analyses were conducted to pool the SMD and associated 95% confidence intervals (CIs) for the continuous data including Lysholm score, WOMAC score, FTA, WBLR, WMJS and MME. The pooled odds ratios (ORs) and associated 95% CIs were used in comparing MMPRT complete healing rates between groups. The pooled rate of MMPRT complete healing was then evaluated following concurrent MMPRT repair during HTO from both case series and comparative studies. The random-effect model was used to account for the effect of between-study heterogeneity and several uncontrolled variables [50]. *I*^2^ statistics were calculated to determine the percentage of total variation attributable to heterogeneity among the included studies. Forest plots were used to graphically present the results of each study and the pooled estimates of the effect size. Descriptive statistics was used for the following variables because of their unsuitability for pooling outcome data: KSKS, KSFS, HSS knee score, IKDC score, Tegner activity scale score, K-L grades, and articular cartilage status.

## Results

### Study selection

**Fig. 1:**
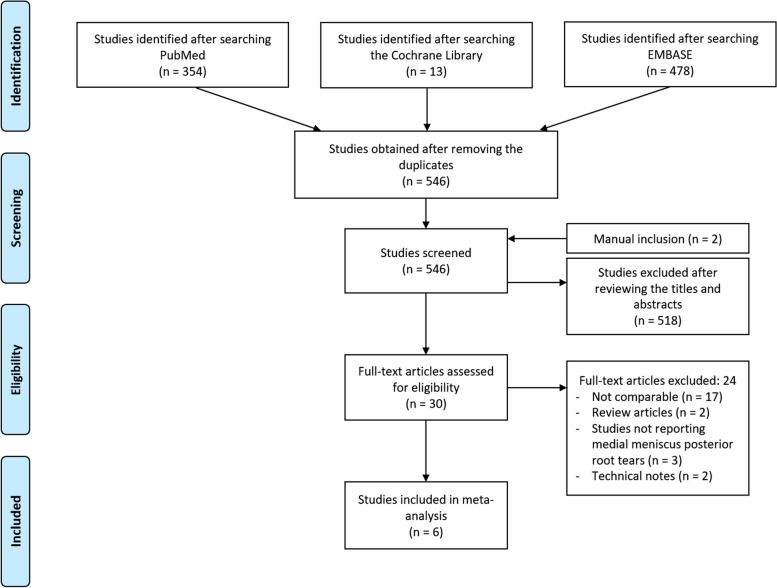
PRISMA flowchart. PRISMA, Preferred Reporting Items for Systematic Reviews and Meta-analyses

Figure 1 shows the flowchart delineating the identification, inclusion, and exclusion of the studies. The inter-reviewer reliability was excellent for both screening (κ = 0.99) and selection of studies (κ = 0.93).

Electronic searches of the PubMed (MEDLINE), EMBASE, and Cochrane Library databases yielded 354, 478, and 13 studies, respectively. A total of 299 duplications were removed, and two publications were added after a manual search, for a total of 548 initial studies. Of these, 518 were excluded after reading the abstracts and full text, and 24 more studies were excluded because of unusable information and inappropriate group comparisons. Thus, a final set of six studies was used in the systematic review and meta-analysis.

### Study characteristics and quality assessment

Four comparative studies and two case series were included [31, 32, 51–54]. The baseline characteristics and patient demographic details are presented in Table 1. The included articles were quite heterogeneous in terms of the baseline characteristics and outcomes of interest. The κ value for data extraction ranged from 0.99 to 1.00. The quality assessment results of the included studies are summarized in Table 2. The κ value for quality assessment ranged from 0.87 to 1.00. In terms of bias among the four comparative studies, two were considered low-risk, with overall scores of 7 stars [51, 53], while the other two were considered moderate-risk, with overall scores of 6 stars [32, 54]. Both case series were considered low-risk, except for the pre-specification of the intervention effect.

**Table 1:** Baseline characteristics of the included studies

Author	Publication year	Study design (level of evidence)	Journal	Case (sample size)	Control (sample size)	Sex (M/F)		Average age (years)		Mean follow-up (years)		Osteotomy type
						Case	Control	Case	Control	Case	Control	
Jing et al	2019	Case-series study (IV)	*Journal of Orthopaedic Surgery*	HTO with MMPRT all-inside repair (27)		12/15		55		1.5		MOWHTO
Lee et al	2019	Comparative study (III)	*Journal of Knee Surgery*	HTO with MMPRT all-inside repair (25)	HTO alone (32)	8/18	10/24	58	60	1.9	2.2	MOWHTO
Ke et al	2020	Comparative study (II)	*Knee Surgery, Sports Traumatology, Arthroscopy*	HTO with MMPRT all-inside repair (30)	HTO alone (34)	4/26	8/26	55	55	2.4	2.5	MOWHTO
Kim et al	2020	Case-series study (IV)	*Knee Surgery, Sports Traumatology, Arthroscopy*	HTO with MMPRT pull-out repair (17)		2/15		51.5		5.5		MOWHTO
Lee et al	2020	Comparative study (III)	*Arthroscopy*	HTO with MMPRT all-inside repair (24) HTO with MMPRT pull-out repair (25)	HTO alone (22)	All-inside repair: 1/23 Pull-out repair: 2/23	2/20	All-inside repair: 57 Pull-out repair: 54	57	All-inside repair: 2.2 Pull-out repair: 2.3	2.4	MOWHTO
Suh et al	2020	Comparative study (III)	*Indian Journal of Orthopaedics*	HTO with MMPRT all-inside repair (43)	HTO alone (38)	8/35	8/30	55.7	56.2	2	2	MOWHTO

*HTO* high tibial osteotomy, *MOWHTO* medial open-wedge high tibial osteotomy, *MMPRT* medial meniscal posterior root tear

**Table 2:** Quality assessment of the included studies

Newcastle–Ottawa assessment scale^a^											
Year	Author	Study design (level of evidence)	Selection				Comparability		Outcome		
			Representativeness of the exposed cohort	Selection of nonexposed cohort	Ascertainment of exposure	Demonstration that the outcome of interest was not present at the start of study	Controlled for age	Controlled for sex, body mass index, or preoperative KL grade	Assessment of outcome	Sufficiency of follow-up	Adequacy of follow-up
2019	Lee et al	Comparative study (III)	★		★	★			★	★	★
2020	Ke et al	Comparative study (II)	★	★	★	★			★	★	★
2020	Suh et al	Comparative study (III)	★		★	★			★	★	★
2020	Lee et al	Comparative study (III)	★	★	★	★			★	★	★
**Effective Practice and Organization of Care assessment**^**b**^											
**Year**	**Author**	**Study design (level of evidence)**	**(1)**	**(2)**	**(3)**	**(4)**	**(5)**		**(6)**	**(7)**	
2019	Jing et al	Case series (IV)	Low	High	Low	Low	Low		Low	Low	
2020	Kim et al	Case series (IV)	Low	High	Low	Low	Low		Low	Low	

^a^Low risk: ≥ 7 stars; Moderate risk: 4–6 stars; High risk: < 4 stars

^b^ (1) independence, (2) pre-specification of the intervention effect, (3) effect of the intervention on data collection, (4) knowledge of the allocated intervention, (5) incomplete outcome data, (6) selective outcome reporting, (7) other risks of bias

### Clinical outcomes

**Fig. 2:**
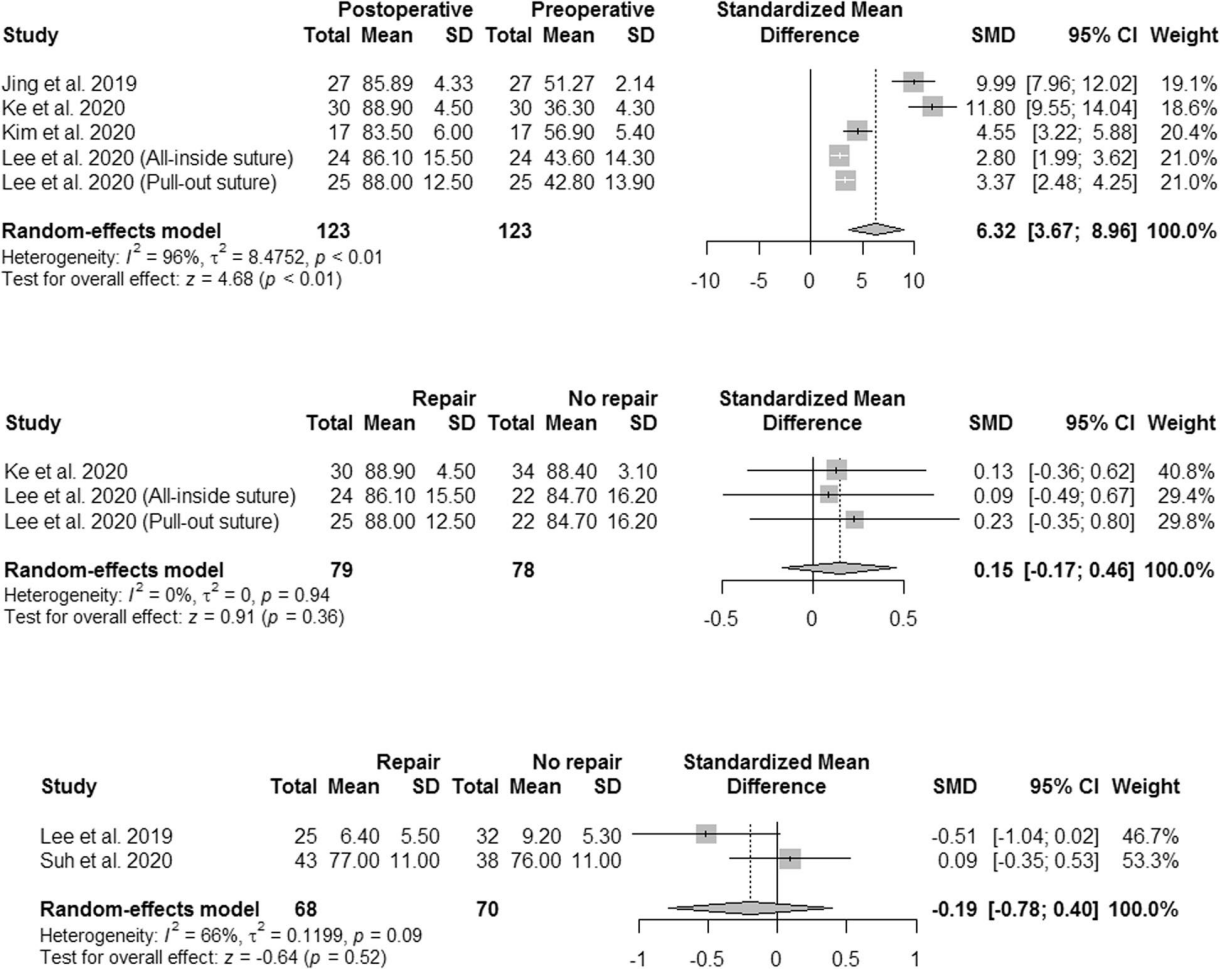
Forest plots showing the treatment effect on the Lysholm score after concurrent MMPRT repair and comparison of the postoperative Lysholm and WOMAC scores between the groups. CI, confidence interval; MMPRT, medial meniscal posterior root tear; SD, standard deviation; SMD, standardized mean difference; Western Ontario and McMaster University (WOMAC)

The clinical outcomes are summarized in Fig. 2 and Table 3. Significant clinical improvement was observed after HTO with concurrent MMPRT repair, with respect to HSS knee score, Lysholm scores, KSKS, KSFS, WOMAC, Tegner score, and IKDC scores [31, 32, 51–53]. A total of five subgroups in four studies reported the preoperative and postoperative Lysholm scores after HTO with concurrent MMPRT repair [31, 51–53]. The overall SMD was estimated at 6.32 (95% CI, 3.67–8.96) (Fig. 2). However, significant heterogeneity was observed (I^2^ = 96%; P < 0.01).

**Table 3:** Clinical and radiological outcomes of the included studies

Author	Publication year	Case (sample size)	Clinical results		Radiological results	
			Case	Control	Case	Control
Jing et al	2019	HTO with MMPRT all-inside repair (27)	HSS: 45.3 to 84.2 Lysholm score: 51.3 to 85.9		FTA: 178 to 186.7 WBLR: 30.1 to 60.6	
Lee et al	2019	HTO with MMPRT all-inside repair (25)	WOMAC (total): 41.4 to 6.4 KSKS: 46.6 to 46.6 KSFS: 57.6 to 91.8 (no significant intergroup difference in all preoperative and postoperative measurements)	WOMAC (total): 40.2 to 9.2 KSKS: 53.2 to 53.2 KSFS: 57.6 to 89.0	FTA: 173.7 to 181.9 WBLR: 21.9 to 64.1 WMJS: 3.4 to 3.7 K-L grade (1/2/3/4): 0/0/16/9 to 0/5/16/4 (no significant intergroup difference in all preoperative and postoperative measurements)	FTA: 173.6 to 181.8 WBLR: 22.2 to 62.0 WMJS: 3.7 to 3.8 K-L grade (1/2/3/4): 0/0/25/7 to 0/2/28/2
Ke et al	2020	HTO with MMPRT all-inside repair (30)	HSS: 38.5 to 85.3 Lysholm score: 36.3 to 88.9 (no significant intergroup difference in all preoperative and postoperative measurements)	HSS: 37.7 to 84.1 Lysholm score: 35.7 to 88.4	FTA: 176.7 to 183.9 WBLR: 25.8 to 62.6 WMJS: 3.8 to 3.7 K-L grade (1/2/3/4): 0/8/20/2 to 0/14/16/0 (no significant intergroup difference in all preoperative and postoperative measurements)	FTA: 176.7 to 183.8 WBLR: 27.8 to 61.7 WMJS: 3.4 to 3.5 K-L grade (1/2/3/4): 0/12/20/2 to 0/19/13/2
Kim et al	2020	HTO with MMPRT pull-out repair (17)	Lysholm score: 56.9 to 83.5 HSS: 56.1 to 81.7		FTA: 174.0 to 179.1 K-L grade (1/2/3/4): 9/8/0/0 to 3/13/1/0	
Lee et al	2020	HTO with MMPRT all-inside repair (24) HTO with MMPRT pull-out repair (25)	All-inside repair group Lysholm score: 43.6 to 86.1 IKDC: 37.9 to 79.8 Tegner score: 3.6 to 4.9 Pull-out repair group Lysholm score: 42.8 to 88.0 IKDC: 38.5 to 81.2 Tegner score: 3.8 to 5.2 (no significant intergroup difference in all preoperative and postoperative measurements)	Lysholm score: 44.4 to 84.7 IKDC: 37.3 to 77.3 Tegner score: 3.9 to 5.1	All-inside repair group FTA: 173.8 to 182.3 WMJS: 2.4 to 2.5 K-L grade (1/2/3/4): 0/0/20/4 to 0/0/21/3 Pull-out repair group FTA: 174.3 to 182.2 WMJS: 2.2 to 2.8 K-L grade (1/2/3/4): 0/0/23/2 to 0/2/21/2 (no significant intergroup difference in all preoperative and postoperative measurements)	FTA: 173.6 to 182.5 WMJS: 2.4 to 2.6 K-L grade (1/2/3/4): 0/0/21/1 to 0/0/20/2
Suh et al	2020	HTO with MMPRT all-inside repair (43)	Postoperative WOMAC: 77	Postoperative WOMAC: 76	FTA: 173.2 to 181.3 WMJS: 3.2 to 3.5	FTA: 172.4 to 180.9 WMJS: 3.6 to 3.6

*HTO* high tibial osteotomy, *MMPRT* medial meniscal posterior root tear, *HSS* Hospital for Special Surgery score, *FTA* femorotibial angle, *WBLR* weight-bearing line ratio, *WOMAC* Western Ontario and McMaster University score, *KSKS* Knee Society Knee Score, *KSFS* Knee Society functional score, *WMJS* width of medial joint space, *K-L* Kellgren–Lawrence, *IKDC* International Knee Documentation Committee subjective knee score

Three subgroups in two studies compared the postoperative Lysholm scores between HTO alone and HTO with concurrent MMPRT repair [51, 53], and two subgroups in two studies compared the postoperative WOMAC scores between HTO alone and HTO with concurrent MMPRT repair [32, 54]. As shown in Fig. 2, the pooled results indicated that concurrent MMPRT repair did not improve postoperative Lysholm and WOMAC scores.

### Radiological outcomes

**Fig. 3:**
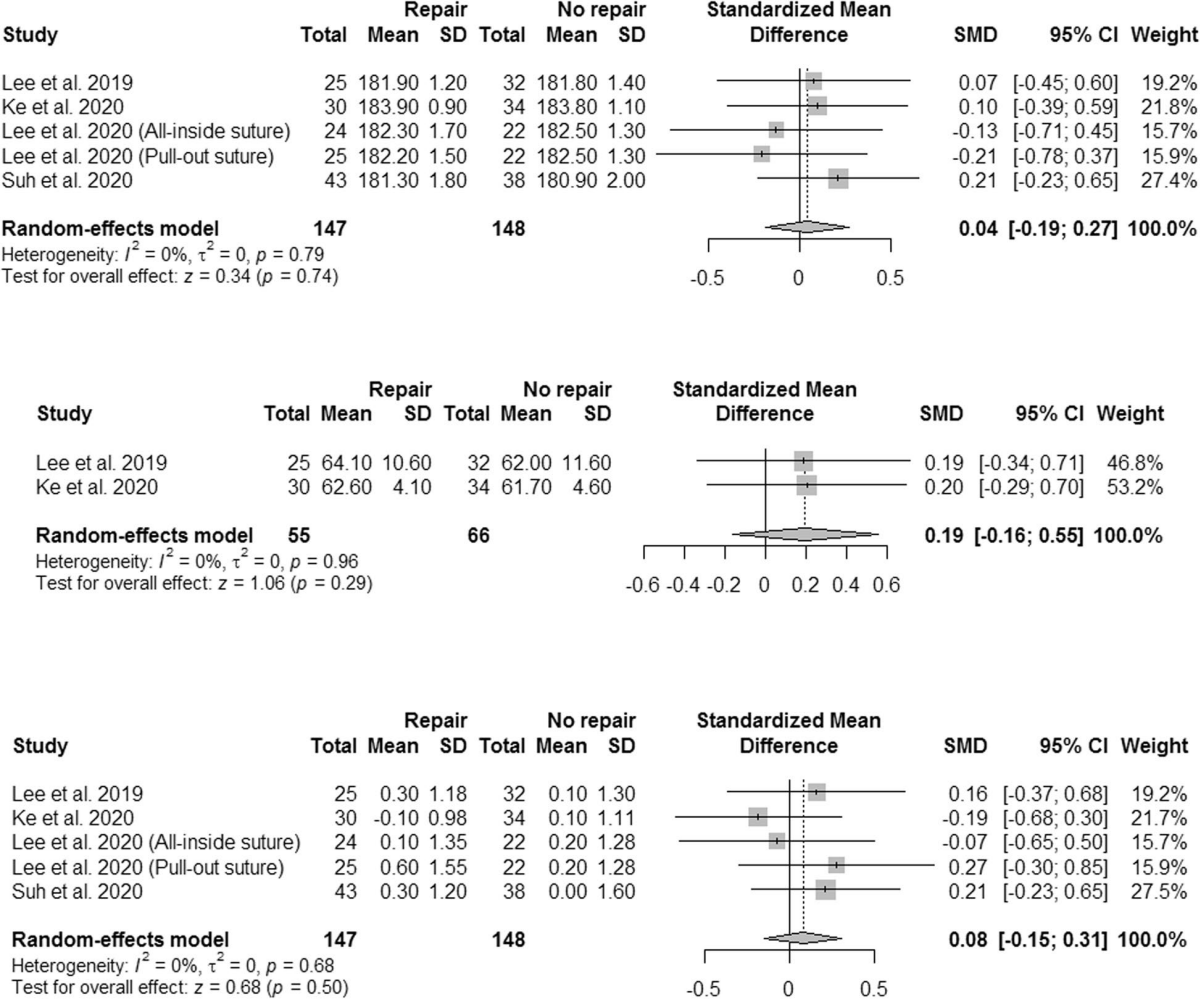
Forest plots showing the intergroup comparisons of the postoperative femorotibial angle (FTA), weight-bearing line ratio (WBLR), and changes in the width of the medial joint space (WMJS). CI, confidence interval; SD, standard deviation; SMD, standardized mean difference

The radiological outcomes, including the FTA, WBLR, WMJS, and K-L grades, are summarized in Fig. 3 and Table 3. A total of five subgroups in four studies reported the postoperative FTAs and changes in the WMJS [31, 51, 53, 54]. The pooled results showed no significant intergroup differences with respect to postoperative FTA and changes in the WMJS. Furthermore, postoperative WBLR did not differ between the two groups (Fig. 3). Three studies compared the preoperative and postoperative K-L grades between HTO alone and HTO with concurrent MMPRT repair [32, 51, 53]. No significant preoperative and postoperative intergroup differences were found.

### Meniscal healing

**Fig. 4:**
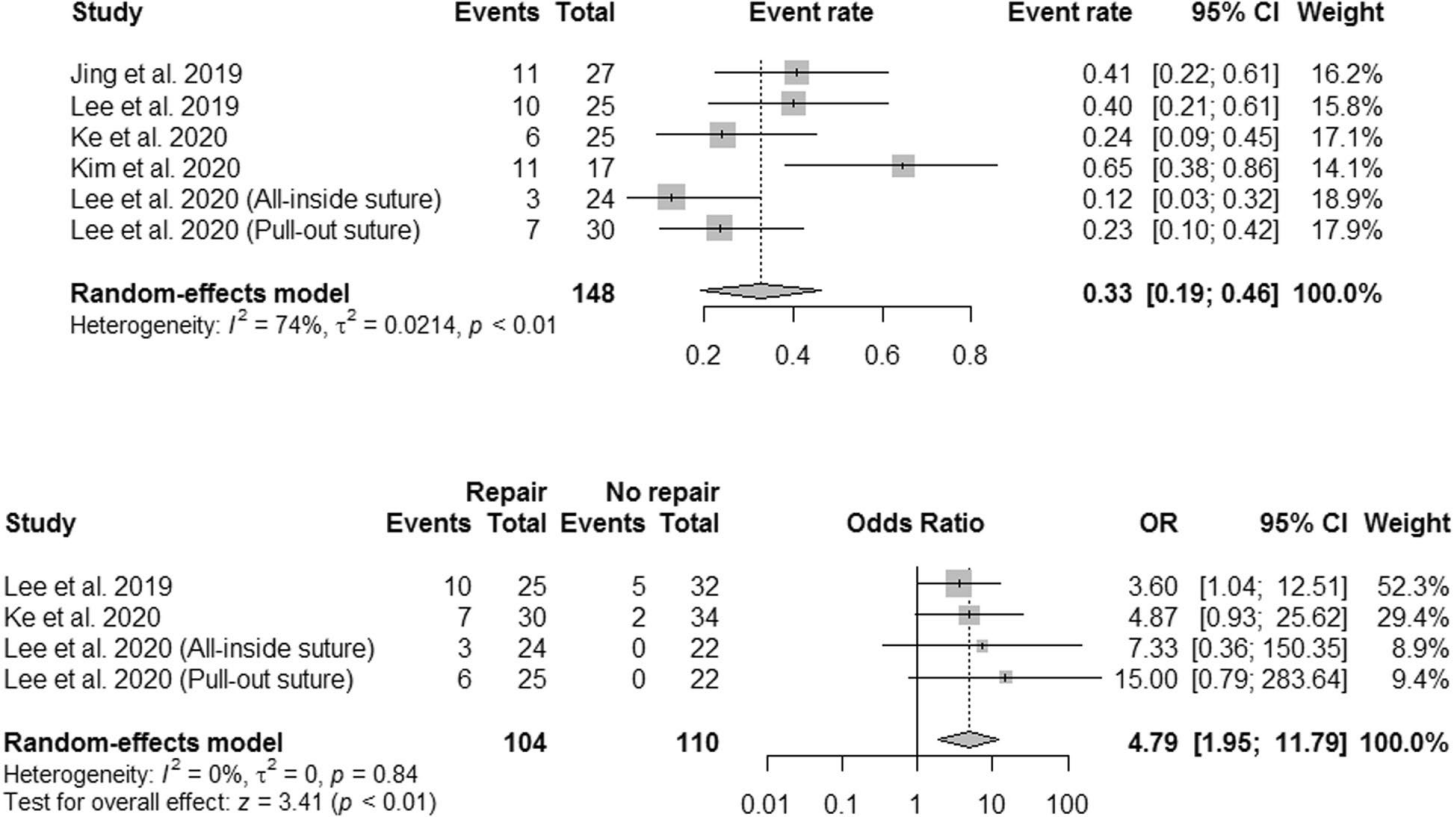
Forest plots showing the treatment effect on meniscal healing after concurrent medial meniscus posterior root tear repair and comparing meniscal healing between groups. CI, confidence interval; SD, standard deviation; SMD, standardized mean difference

The results of meniscal healing are presented in Table 4 and Fig. 4. A total of six subgroups in five studies reported the second-look arthroscopic findings on MMPRT healing status [31, 32, 51–53]. The pooled event rate for complete healing of the medial meniscus posterior root was 0.33 (95% CI, 0.19–0.46) (Fig. 4). However, significant heterogeneity among the studies was observed (I^2^ = 74%; P < 0.01).

**Table 4:** Medial meniscal extrusion and arthroscopic findings of the included studies

Author	Publication year	Case (sample size)	Medial meniscal extrusion		Meniscal healing		Articular cartilage	
			Case	Control	Case	Control	Case	Control
Jing et al	2019	HTO with MMPRT all-inside repair (27)			Meniscal complete healing: 11/27 (40.7%)		Complete cartilage coverage: 27/27 (100%)	
Lee et al	2019	HTO with MMPRT all-inside repair (25)	4.6 to 4.5 (no intergroup difference preoperatively and postoperatively)	4.3 to 4.5	Meniscal complete healing: 10/25 (40%) (significantly higher in case group)	Meniscal complete healing: 5/32 (15.6)	ICRS grade of the MFC (0/1/2/3/4): 0/0/4/9/12 to 1/3/6/9/6 (no intergroup difference preoperatively and postoperatively)	ICRS grade of the MFC (0/1/2/3/4): 0/1/8/12/14 to 2/6/9/11/7
Ke et al	2020	HTO with MMPRT all-inside repair (30)	4.1 to 4.0 (no intergroup difference preoperatively and postoperatively)	4.2 to 4.2	Meniscal complete healing: 7/30 (23.3%) (significantly higher in case group)	Meniscal complete healing: 2/34 (5.9%)	Outerbridge grade of medial compartment (1/2/3/4): 0/4/14/12 to 0/8/18/4 (no intergroup difference preoperatively and postoperatively)	Outerbridge grade of medial compartment (1/2/3/4): 0/6/16/12 to 0/10/18/6
Kim et al	2020	HTO with MMPRT pull-out repair (17)	3.0 to 3.1		Meniscal complete healing: 11/17 (64.7%)		Outerbridge grade of MFC (1/2/3/4): 5/4/7/0 to 6/9/2/0	
Lee et al	2020	HTO with MMPRT all-inside repair (24) HTO with MMPRT pull-out repair (25)			All-inside repair group Meniscal good healing: 3/24 (12.5%) (significantly higher compared with control group) Pull-out repair group Meniscal good healing: 6/25 (24%) (significantly higher compared with control group)	Meniscal good healing: 0/22 (0%)	All-inside repair group Preoperative ICRS grade of the MFC and MTP (1/2/3/4): 0/6/13/5 and 3/9/11/1, respectively ICRS regeneration grade of the MFC and MTP (excellent/good/poor): 4/12/8 and 2/14/8, respectively Pull-out repair group Preoperative ICRS grade of the MFC and MTP (1/2/3/4): 0/5/13/7 and 3/9/11/2, respectively ICRS regeneration grade of the MFC and MTP (excellent/good/poor): 6/14/5 and 3/14/8, respectively	Preoperative ICRS grade of the MFC and MTP (1/2/3/4): 0/5/11/6, 2/8/10/2, respectively ICRS regeneration grade of the MFC and MTP (excellent/good/poor): 3/11/8, 2/10/10, respectively (no intergroup difference preoperatively and postoperatively)

*HTO* high tibial osteotomy, *MMPRT* medial meniscal posterior root tear, *ICRS* International Cartilage Repair Society, *MFC* medial femoral condyle, *MTP* medial tibial plateau

Four subgroups of three studies were used to compare the MMPRT complete healing rates [32, 51, 53]. The pooled results indicated that the MMPRT complete healing rate was higher with concurrent MMPRT repair (OR, 4.79; 95% CI, 1.945–11.79; P < 0.01) (Fig. 4).

### Amount of MME

**Fig. 5:**
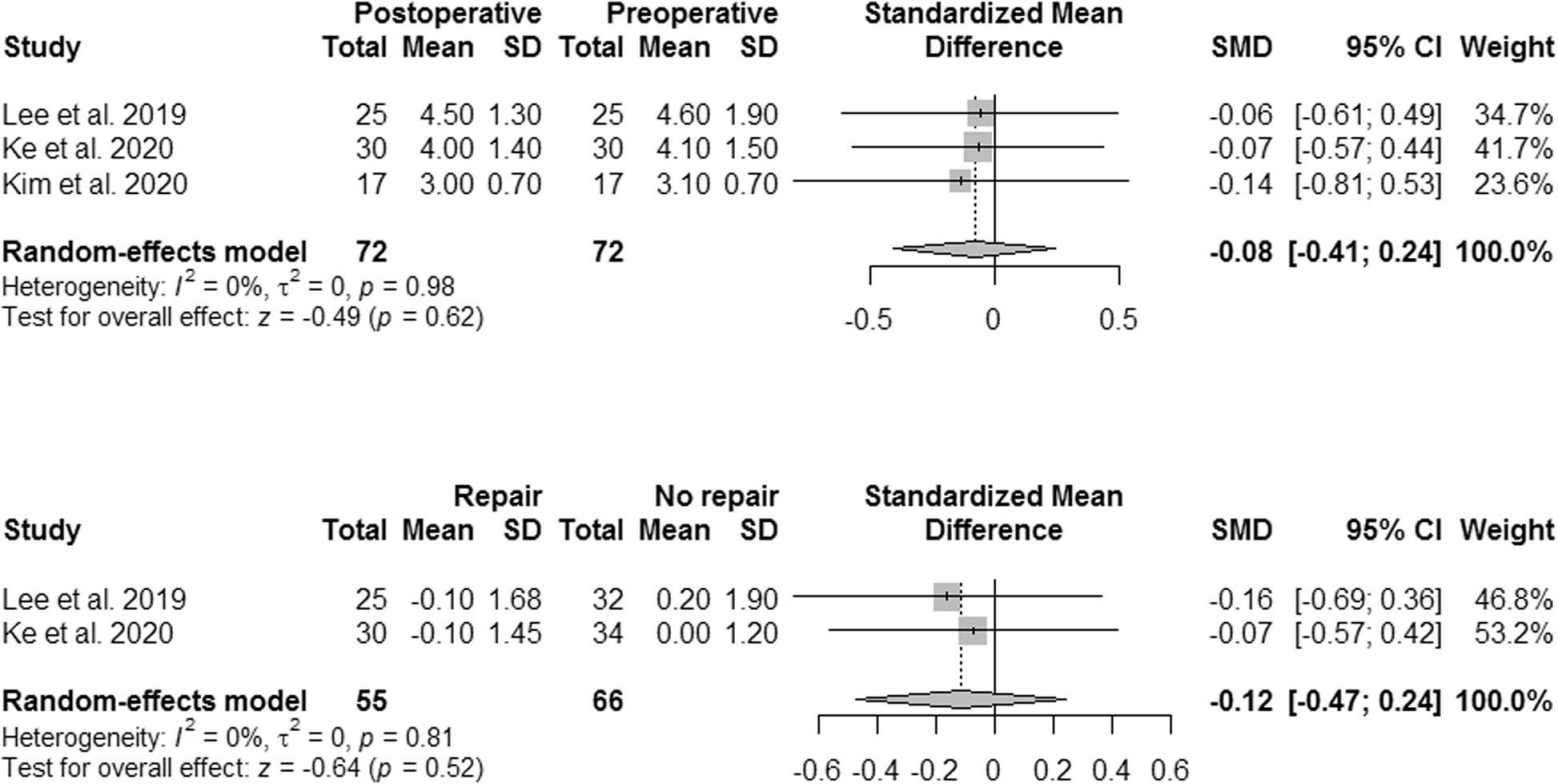
Forest plots showing the treatment effect on the medial meniscus extrusion after concurrent medial meniscus posterior root tear repair and comparison of the medial meniscus extrusion between the groups. CI, confidence interval; SD, standard deviation; SMD, standardized mean difference

Three studies reported the amount of MME, and the results are summarized in Table 4 [32, 51, 52]. The pooled treatment effects showed no significant difference in the preoperative and postoperative MME after HTO with concurrent MMPRT repair (Fig. 5), as well as no significant difference in the changes in the MME between patients with and without concurrent MMPRT repair (SMD, -0.12; 95% CI, -0.47–0.24; P = 0.52; Fig. 5).

### Articular cartilage findings

Second-look arthroscopic findings of articular cartilage are summarized in Table 4. Five included studies reported the cartilage status evaluated with second-look arthroscopy [31, 32, 51–53]. Articular cartilage status was reported using the ICRS grading system [32], as well as the Outerbridge grading system, both preoperatively and postoperatively (Table 4) [51, 52]. Lee et al. reported cartilage status using both ICRS degeneration and regeneration grading systems (Table 4) [53].

## Discussion

Our results suggest that concurrent MMPRT repair during HTO improves MMPRT healing, based on second-look arthroscopic findings, and subjective postoperative patient scores. However, no additional beneficial effect on cartilage status and subjective patient and radiological outcomes was observed with concurrent MMPRT repair during HTO compared to HTO alone during short-term follow-up. Therefore, to date, concurrent MMPRT repair is considered unnecessary, owing to the lack of evidence on outcome benefits.

We investigated the effect of concurrent repair of MMPRTs based on the following three questions: (1) “Does it improve clinical and radiological outcomes?”, (2) “Does it improve the rate of complete healing of the medial meniscus?”, and (3) “Does it improve cartilage status based on second-look arthroscopic findings?”.

Clinical improvements in knees with medial OA and varus malalignment can be achieved by increasing the medial proximal tibial angle and reducing one-sided medial compartment overload. Although the loss of meniscal hoop tension results in a decrease in the tibiofemoral contact area, varus malalignment of the lower limb or a lower medial proximal tibial angle is more important in joint deterioration with increased tibiofemoral pressure in the affected compartment [6–8]. Therefore, the transfer of the weight-bearing line into the lateral compartment and increase of medial proximal tibial angle after HTO alone can lead to adequate unloading in the affected compartment and significant clinical improvement [13, 55, 56]. Additional benefits of concurrent MMPRT repair were not demonstrated during the short-term follow-up.

The progression of OA and loss of the correction angle with recurrence of varus deformity mainly account for the progression of clinical and radiological deterioration in HTO over time [12, 57]. However, the results of the present review showed no significant difference in the postoperative FTA, WBLR, changes in the WMJS, and K-L grade during approximately 2 years of follow-up, regardless of whether meniscal repair was performed concurrently. Therefore, although a long-term follow-up was not employed, concurrent meniscal repair may be considered unnecessary to obtain good short-term results, owing to the limited outcome benefits observed.

Nevertheless, concurrent MMPRT repair during HTO may improve the healing process of the medial meniscus. Physiological tensile strain might be important for activating extracellular matrix production in meniscal horn cells [58]. This supports the hypothesis that MMPRT repair can change the composition of the medial meniscus and suppress degeneration by improving the meniscal hoop tension [59]. According to the arthroscopic visual classification of healed MMPRTs, the rate of complete healing was higher in those who underwent concurrent MMPRT repair [21–23]. However, the MMPRT complete healing rate was very low, with a pooled rate of 33% (95% CI, 19%-46%), and there was significant heterogeneity among studies. Because only patients with K-L grade < 3 were included, Kim et al. reported a high rate of complete healing of MMPRTs compared to other studies [52]. Furthermore, the extruded meniscus was not reduced in terms of MME after MMPRT repair. Restoration of hoop tension depends on actual healing in a reduced position, and if the meniscus remains extruded, it is unlikely that restoration of hoop tension has occurred [60]. Therefore, concurrent MMPRT repair during HTO might not optimize the knee joints in terms of improved tibiofemoral contact surface and restoration of hoop tension despite visual meniscal healing [60–63].

Owing to the heterogeneity in the evaluating the articular cartilage, it was difficult to perform a pooled analysis. Most second-look arthroscopic findings showed no difference in cartilage recovery between patients with and without concurrent MMPRT repair. Although the evaluation method was not described, Jing et al. reported that all patients showed complete coverage of the preoperative cartilage defects in the medial femoral condyles on second-look arthroscopy [31]. Kim et al. reported improved Outerbridge grades of the medial femoral condyle after HTO with concurrent MMPRT repair [52]. Lee et al. and Ke et al. reported favorable medial compartment coverage with no significant intergroup difference assessed by ICRS grading and Outerbridge grading systems, respectively [32, 51]. Furthermore, Lee et al. reported no significant intergroup difference of cartilage recovery in medial compartment assessed by ICRS regeneration grading system [53] As long-term benefits are the most important and ultimate goal of joint preservation surgery, results on cartilage recovery and disease progression following concurrent MMPRT repair during HTO should be reassessed in a long-term follow-up period.

This review has some limitations. First, only a small number of studies with short-term follow-up and low levels of evidence were analyzed. Second, the studies were greatly heterogeneous, regarding the study design, baseline characteristics, assessment methods, and outcomes such as different preoperative K-L grades and population sex distribution. Third, the MMPRT repair techniques varied among the studies, which could have also caused their heterogeneity. It was not possible to analyze technique-specific efficacy due to the small allocated sample size. Fourth, there might be potential biases on second-look arthroscopic findings, such as the healing status of MMPRTs and recovery of articular cartilage, compared to other quantitative results. Fifth, a definite conclusion cannot be drawn because of the lack of long-term results. Well-organized comparative studies with long-term follow-up and larger sample sizes are required to establish the definite benefits of concurrent MMPRT repair during HTO. Additionally, publication bias was not assessed because to the few number of studies included (< 10) made it difficult to distinguish chance from real bias [64].

## Conclusions

Concurrent MMPRT repair during HTO for medial compartmental OA with MMPRTs has little benefits on clinical, radiological, and arthroscopic outcomes during the short-term follow-up. Further accumulation of evidence is needed for long-term effects.

## Data Availability

The datasets used and/or analyzed during the current study are available from the corresponding author upon reasonable request.

## Abbreviations

FTA: Mechanical femorotibial angle
HTO: High tibial osteotomy
HSS: Hospital for Special Surgery
ICRS: International Cartilage Repair Society
IKDC: International Knee Documentation Committee
K-L: Kellgren–Lawrence
KSKS: Knee Society Knee Score
KSFS: Knee Society Functional Score
MME: Medial meniscus extrusion
MMPRT: Medial meniscal posterior root tear
OA: Osteoarthritis
PRISMA: Preferred Reporting Items for Systematic reviews and Meta-Analyses
SMD: Standardized mean difference
WBLR: Weight-bearing line ratio
WMJS: Width of the medial joint space
WOMAC: Western Ontario and McMaster University
